# Editorial: Metabolic Changes After Kidney Transplantation

**DOI:** 10.3389/fmed.2021.709644

**Published:** 2021-07-08

**Authors:** Ekamol Tantisattamo, Bing T. Ho, Biruh T. Workeneh

**Affiliations:** ^1^Harold Simmons Center for Kidney Disease Research and Epidemiology, Division of Nephrology, Hypertension and Kidney Transplantation, Department of Medicine, University of California, Irvine School of Medicine, Orange, CA, United States; ^2^Nephrology Section, Department of Medicine, Tibor Rubin Veterans Affairs Medical Center, Veterans Affairs Long Beach Healthcare System, Long Beach, CA, United States; ^3^Multi-Organ Transplant Center, Section of Nephrology, Department of Internal Medicine, William Beaumont Hospital, Oakland University William Beaumont School of Medicine, Royal Oak, MI, United States; ^4^Comprehensive Transplant Center, Division of Nephrology and Hypertension, Department of Medicine, Northwestern University Feinberg School of Medicine, Chicago, IL, United States; ^5^Section of Nephrology, University of Texas MD Anderson Cancer Center, Houston, TX, United States

**Keywords:** chronic kidney disease-mineral bone disorders, electrolyte disturbances, hypertension, living donor, metabolic changes, metabolic syndrome, kidney transplantation, nutrition

Kidney transplantation is the treatment of choice for those nearing or suffering from end-stage kidney disease (ESKD) (1). Transplantation provides a significant survival benefit, restoring kidney glomerular filtration and homeostatic functions, including fluid and electrolyte balance. Furthermore, it improves the quality of life, freeing patients from the obligation to have regular dialysis. Despite all the articulated benefits of kidney transplant recipients, the cardiovascular and overall mortality risk remains high compared to the general population (2). Indeed cardiovascular diseases (CVD) occur at high rates and are the most common cause of mortality in kidney transplant recipients (3). Both traditional and non-traditional risk factors of CVD in kidney transplant recipients contribute to morbidity and mortality. Some risk factors are modifiable, such as diet or behavioral, but other determinants are difficult or impossible to modify. In addition to describing various metabolic challenges associated with kidney transplantation, the article series explores the opportunity to intervene and improve kidney allograft and patient outcomes.

Metabolic alteration and chronic inflammation appear early in chronic kidney disease (CKD) stages and progress through ESKD, making CKD one of the most important cardiovascular risk factors. After successful kidney transplantation, these changes may become normalized or be persist. In this timely topic of metabolic changes after kidney transplantation, five articles address various topics related to common metabolic changes post-kidney transplantation, including nutrition for kidney transplant recipients, electrolytes and acid-base disturbances, chronic kidney disease-mineral bone disorders (CKD-MBD), and post-transplant hypertension. Since successful kidney transplantation also depends on kidney donor factors, an article related to inflammatory change and biomarkers in living kidney donors was highlighted.

Diet and nutrition play an essential role in the slow progression of CKD (4). Likewise, nutrition in kidney transplantation is one of the critical components in caring for kidney transplant recipients to slow the progression of kidney allograft function and mitigate poor transplant outcomes. Despite evidence that has shown the benefit of dietary intervention on kidney health, particularly dietary protein intake in the CKD population (4–6), there is a lack of studies regarding dietary interventions to slow the progression of kidney allograft function (7–12). In addition, since kidney transplantation involves the dynamic process from pre-, peri-, and post-transplant periods when physical stress and intensity of immunosuppressive medications have been changed, extrapolating data or clinical practice guidelines from general, CKD, or ESKD populations may not be ideal. Nolte Fong and Moore reviewed evidence related to nutritional complications after kidney transplantation, both macronutrients including fat, protein, carbohydrate, and micronutrients, including phosphorus, magnesium, vitamin D. Nutrition trends, dietary intervention, and recommended diet intervention in kidney transplantation were discussed.

Electrolyte and acid-base disturbances are prevalent in kidney transplant recipients, and the pathogenesis of some of these are different from those in CKD. Although regaining kidney function but possibly at the suboptimal level in immunosuppressive milieu, some electrolyte disturbances are almost universal. Pathogenesis of post-transplant electrolyte and acid-base imbalances involve the interplay between non-immunologic and immunologic factors. The non-immunological factors can be persistent from pre- through post-transplant periods, whereas immunological factors occurring post-transplant result primarily from immunosuppressive medications. Pochineni and Rondon-Berrios presented an excellent review of electrolyte and acid-base disorders commonly seen in kidney transplant recipients, including hyperkalemia, metabolic acidosis, hypercalcemia, hypomagnesemia, and hypophosphatemia. The epidemiology, pathogenesis, and clinical manifestations were reviewed, and recommendations for management were summarized.

CKD-MBD is one of the fascinating metabolic changes after successful kidney transplantation, and there has been a rapid growth of evidence in kidney transplantation. Laboratory components, bone disorders, and tissue calcification may not be reversible after kidney transplantation. Even if they are reversed toward normalized values or status during non-CKD, the pattern can vary widely. Vangala et al. provided a comprehensive review in all clinically relevant aspects of CKD-MBD after kidney transplantation, including the pathophysiology of CKD-MBD from pre- through post-transplantation, clinical presentations, detailed and separated disorders of bone and mineral metabolism related to CKD-MBD including calcium, phosphorus, parathyroid hormone, and vitamin D, common bone diseases in CKD-MBD including renal osteodystrophy, osteopenia, osteoporosis, and osteomalacia. Investigations for biochemical markers of CKD-MBD and bone health assessment, as well as pharmacological and surgical management for CKD-MBD, were discussed.

Hypertension is one of the clusters of metabolic disorders in metabolic syndrome (13) and one of the most common but modifiable risk factors for CVD (14). From the advanced stage of CKD to post-transplant periods, hypertension is almost universal. Contributing factors of post-transplant hypertension involve both factors resulted from complications of ESKD and transplant-related factors. The ESKD-related factors include volume overload, stimulated renin-angiotensin-aldosterone system, sympathetic nervous system activation, arterial stiffness, vascular endothelial dysfunction, sleep apnea, erythropoiesis-stimulating agents (15). After successful kidney transplantation, both non-immunological factors, which endure from the ESKD period, and immunological factors, particularly associated with immunosuppressive medications, contribute to post-transplant hypertension. Tantisattamo et al. reviewed several aspects of post-transplant hypertension, including epidemiology, pathogenesis primarily related to immunosuppression. Obtaining an accurate blood pressure measurement and reading was emphasized by using 24-h ambulatory blood pressure monitoring. The advantages and disadvantages of different types of commonly used antihypertensive medications in the setting of immunosuppression and potential electrolyte and acid-base disturbances were compared and contrasted. Well-recognized but under-diagnosed causes of post-transplant hypertension, including transplant renal artery stenosis and obstructive sleep apnea, were reviewed. Non-pharmacological management for uncontrolled post-transplant hypertension, including renal nerve denervation and native nephrectomy, were discussed.

Although the metabolic changes in kidney transplant recipients, including nutrition-related changes, acid-base disturbances, CKD-MBD, and hypertension, as non-immunological factors, contribute to poor transplant outcomes both kidney allograft and patient survivals, these factors interconnect with immunological factors (Table 1 and Figure 1). Therapy of modifying immunosuppressive medications can be limited from potentially adverse kidney allograft outcomes; several interventions for non-immunological factors are available with varied effectiveness. While medical therapies are the most frequently utilized option and improve or normalize acid-base, electrolytes, MBP markers, long-term transplant outcomes may not be different from non-medical therapies, but potential medication side effects may occur. Dietary modification is beneficial and supplementary for the medical therapies for all post-transplant electrolyte and acid-base disturbances and hypertension. Furthermore, surgical and procedural interventions for post-transplant CKD-MBD and hypertension are options if medical or dietary interventions are ineffective, although data regarding long-term transplant outcomes are required (Figure 1).

**Table 1:** Prevalence and pathogenesis of metabolic changes after kidney transplantation.

	**Prevalence**	**Pathogenesis**	
		**Non-immunologic**	**Immunologic**
Metabolic acidosis	•12–58% (16, 17) •13–16% at 1-year post-transplant (16, 18)	•Decreased Nephron mass (19) •Ischemic tubular dysfunction •Eubicarbonatemic metabolic acidosis (20)	•Immunosuppressive medications (21) •RTA •Type 1: CsA •Type 2: Tacrolimus •Type 4: TMP/SMX •Diarrhea: –Mycophenolate –CMV colitis
Hyperkalemia	•8.8% for patients on CNIs with stable kidney allograft function (22) •9.6% during the first 6 months post-transplant (23)	•RTA •RAAS blockade •ENaC blockade: •TMP/SMX •Pentamidine •Insulinopenia or insulin resistance leads to Intracellular K shift (24)	•CNIs inhibit mineralocorticoid receptor transcriptional activity and lead to impaired mineralocorticoid function and aldosterone resistance (25, 26) •Tacrolimus activates the thiazide-sensitive sodium-chloride cotransporter in the distal convoluted tubule (27)
Hypercalcemia	•10 to 59% (28, 29) •5–10% after a 1-year post-translant (30–32)	•Tertiary hyperparathyroidism •Increased calcitriol production •Increased PTH •Hypophosphatemia •Soft-tissue calcium phosphate resorption (28)	•Steroids (28)
Hypomagnesemia	•6.6% treated with tacrolimus and 1.5% treated with cyclosporine (33) •20% persist several years post-transplant (34) •22.4% up to 6 years post-transplant (35)	•Hyperglycemia (Pochineni and Rondon-Berrios) •Diuretics •Proton pump inhibitors	•CNI causes distal Mg excretion by decreasing Mg transporter TRPM6 in the distal tubule (36) •CsA downregulates renal epidermal growth factor production (37) •Steroids (Pochineni and Rondon-Berrios)
Hypophosphatemia	•42% at 30 days post-transplant (38) •40–93% (Pochineni and Rondon-Berrios; Vangala et al.)	•Persistent elevated FGF23, PTH, and other phosphatonins (39, 40) •Increased dialysis vintage (41) •Decreased calcitriol levels	•Tacrolimus decreases phosphate cotransporter NaPi-2a in the proximal tubule and leads to increased renal phosphate wasting (42)
Secondary or tertiary hyperparathyroidism	•25 to>80% after 1-year post-transplant (43–46)	•Parathyroid hyperplasia or adenoma •Allograft CKD	
Hypovitaminosis D	•80.7% (51–60.2%) insufficiency (15-30 ng/mL) and 20.5–29% deficiency (<15 ng/mL) in 25(OH)D (47, 48)	•Decreased sunlight exposure	
*Bone disorders*			
Renal Osteodystrophy	•91.3% (49)	•Allograft CKD	
*High bone turnover states*	•22.8% • 24.6%	•Persistent MBD	
–Mild osteitis fibrosa–Osteitis fibrosa*Low bone turnover states*–Adynamic bone–Osteomalacia*Mixed uremic osteodystrophy*	•5.3% •3.5% • 12.3%		
Osteopenia and Osteoporosis	•50–52.5% •15–27.5% (50)	•Residual MBD •Hypomagnesemia (51) •Increased PTH secretion •Increased osteoclastogenesis •Decreased osteoblast proliferation •Hypogonadism (Vangala et al.)	•Glucocorticoid-induced bone loss (52) •Decreased osteoblast proliferation and differentiation •Increased promoting osteoclastogenesis •Decreased intestinal calcium absorption (53) •Increased urinary calcium loss •Steroid-induced gonadal hormone deficiency •Tacrolimus and sirolimus lead to osteoblast apoptosis (54)
Osteonecrosis	•3–40% (55, 56)		•Glucocorticoid (57) •Decreased vascular endothelial growth factor •Alterations in circulating lipids with resultant fat emboli •Increased apoptosis of osteoblasts, osteocytes and endothelial cells •Adipogenesis, procoagulant state •Modulation of vasoactive mediators •Increased intraosseous pressure leads to ischemia and necrosis
Hypertension	•24–90% (Tantisattamo et al.)	•Volume overload •Pain •Rebound hypertension •Obesity •FGF23 •Transplant renal artery stenosis •Obstructive sleep apnea •Sympathetic over activity	•Immunosuppressive medications •CNIs •Steroids •Hypertensive donor kidneys •Chronic renal allograft dysfunction

*CMV, cytomegalovirus; CsA, cyclosporine; CNI, calcineurin inhibitors; ENaC, epithelial sodium channel; FGF23, fibroblast growth factor 23; K, potassium; PTH, parathyroid hormone; RAAS, renin-angiotensin aldosterone; RTA, renal tubular acidosis; TMP/SMX, trimethoprim/sulfamethoxazole; 25(OH)D, total 25-hydroxy vitamin D*.

**Figure 1:**
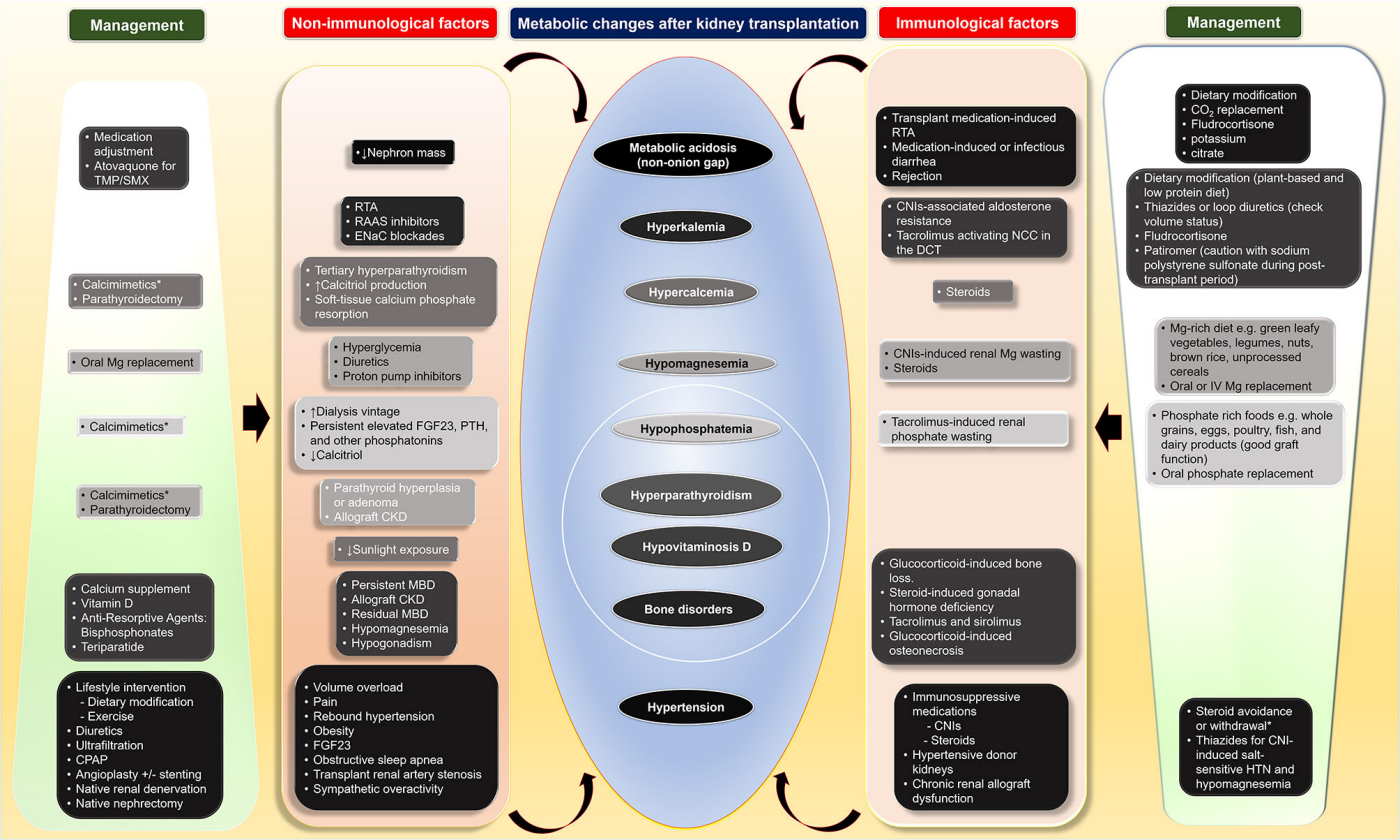
Four main metabolic changes after kidney transplantation as non-immunological factors and their interconnection with immunological factors as well as therapeutic interventions including dietary modification, medical, and surgical treatments. CKD, chronic kidney disease; CNI, calcineurin inhibitors; CO_2_, total carbon dioxide; CPAP, continuous positive airway pressure; DCC, distal convoluted tubule; ENaC; epithelial sodium channel; FGF23, fibroblast growth factor 23; HTN, hypertension; IV, intravenous; MBD, mineral bone disorder; Mg, magnesium; NCC, thiazide-sensitive sodium-chloride cotransporter; PTH, parathyroid hormone; RAAS, renin-angiotensin aldosterone; RTA, renal tubular acidosis; TMP/SMX, trimethoprim/sulfamethoxazole.

Finally, a provocative prospective cohort study by Díaz-de la Cruz et al. investigated the inflammatory state in living kidney donors. Several long-term studies have demonstrated kidney donation safety in carefully selected donors (58, 59). However, there is a small fraction that develops CKD, and it is important to investigate why this happens to increase the safety of donation. The authors examined patterns of pro-inflammatory cytokines and markers of oxidative stress and found significant changes in the oxidative state markers after 6-months post-donation. The study's findings add to what is known about metabolic changes after kidney donation, and additional study is required to characterize alterations further and correlate them with clinical importance.

While improved kidney function after successful kidney transplantation significantly lowers kidney transplant recipients' mortality, profound metabolic alterations affect nutrition, electrolyte, acid-base balance, mineral bone metabolism, and insulin resistance. Metabolic syndrome and post-transplant diabetes are common, and cardiovascular damage resulting from the alterations in metabolism is accelerated, leading to excess mortality compared to the general non-CKD population, which still exist or are partially improved. Uncorrected, these metabolic changes can adversely affect post-transplant kidney allograft and patient outcomes, particularly morbidity and mortality from CVD. Living kidney donation is also the main component of successful kidney transplantation, and further investigation in the file of metabolic and inflammation should be another novel area of research.

## Author Contributions

ET, BH, and BW participated in designing the manuscript's topics and detail, writing the manuscript, and approved the manuscript's submitted version. All authors contributed to the article and approved the submitted version.

## Conflict of Interest

The authors declare that the research was conducted in the absence of any commercial or financial relationships that could be construed as a potential conflict of interest.

## Abbreviations

CMV: cytomegalovirus
CKD: chronic kidney disease
CKD-MBD: chronic kidney disease-mineral bone disorders
CO_2_: total carbon dioxide
CPAP: continuous positive airway pressure
CsA: cyclosporine
CNI: calcineurin inhibitors
CVD: cardiovascular disease
DCC: distal convoluted tubule
ENaC: epithelial sodium channel
ESKD: end-stage kidney disease
FGF23: fibroblast growth factor 23
HTN: hypertension
IV: intravenous
K: potassium
MBD: mineral bone disorder
Mg: magnesium
NCC: thiazide-sensitive sodium-chloride cotransporter
PTH: parathyroid hormone
RAAS: renin-angiotensin aldosterone
RTA: renal tubular acidosis
TMP/SMX: trimethoprim/sulfamethoxazole
25(OH)D: total 25-hydroxy vitamin D.
